# American Pharmaceutical Association

**Published:** 1878-03

**Authors:** 


American Pharmaceutical Association.—We have, during the year not only the meeting of the Medical Association of Georgia, but also that of the "American Pharmaceutical Association," which meets in Atlanta on the first Tuesday in September next.

This organization has been in successful operation for about twenty years, and held its last session in Toronto, Canada, last September. The Association with great unanimity decided to hold the next annual meeting in Atlanta, at the suggestion of our friend, Mr. Ingalls, of Macon, Georgia, who is a member of that body, and who was elected Vice-President of the Association. In the October number of this Journal, a synopsis of the transactions at Toronto was given, and we shall take pleasure in making a full report of the proceedings of the meeting to be held here in September.

The successful treatment of disease by physicians is greatly facilitated by advancement in practical pharmacy, and we take pleasure in promoting, by any means at our command, progress in this useful science.

